# Patterns and risk factors of avian influenza A(H5) and A(H9) virus infection in pigeons and quail at live bird markets in Bangladesh, 2017–2021

**DOI:** 10.3389/fvets.2022.1016970

**Published:** 2022-10-26

**Authors:** Ariful Islam, Shariful Islam, Emama Amin, Rashedul Hasan, Mohammad Mahmudul Hassan, Mojnu Miah, Mohammed Abdus Samad, Tahmina Shirin, Mohammad Enayet Hossain, Mohammed Ziaur Rahman

**Affiliations:** ^1^Centre for Integrative Ecology, School of Life and Environmental Science, Deakin University, Melbourne, VA, Australia; ^2^EcoHealth Alliance, New York, NY, United States; ^3^Institute of Epidemiology, Disease Control and Research (IEDCR), Dhaka, Bangladesh; ^4^One Health Laboratory, International Center for Diarrheal Diseases Research, Bangladesh (icddr, b), Dhaka, Bangladesh; ^5^Queensland Alliance for One Health Sciences, School of Veterinary Science, University of Queensland, Brisbane, QLD, Australia; ^6^National Reference Laboratory for Avian Influenza, Bangladesh Livestock Research Institute (BLRI), Savar, Bangladesh

**Keywords:** avian influenza, seasonality, pigeons, quail, live bird market, biosecurity practices

## Abstract

The avian influenza virus (AIV) impacts poultry production, food security, livelihoods, and the risk of transmission to humans. Poultry, like pigeons and quail farming, is a growing sector in Bangladesh. However, the role of pigeons and quails in AIV transmission is not fully understood. Hence, we conducted this study to investigate the prevalence and risk factors of AIV subtypes in pigeons and quails at live bird markets (LBMs) in Bangladesh. We collected oropharyngeal and cloacal swab samples from 626 birds in 8 districts of Bangladesh from 2017 to 2021. We tested the swab samples for the matrix gene (M gene) followed by H5, H7, and H9 subtypes using real-time reverse transcriptase-polymerase chain reaction (rRT-PCR). We then used exploratory analysis to investigate the seasonal and temporal patterns of AIV and a mixed effect logistic model to identify the variable that influences the presence of AIV in pigeons and quails. The overall prevalence of AIV was 25.56%. We found that the prevalence of AIV in pigeons is 17.36%, and in quail is 38.75%. The prevalence of A/H5, A/H9, and A/H5/H9 in quail is 4.17, 17.92, and 1.67%, respectively. Furthermore, the prevalence of A/H5, A/H9, and A/H5/H9 in pigeons is 2.85, 2.59, and 0.26%. We also found that the prevalence of AIV was higher in the dry season than in the wet season in both pigeons and quail. In pigeons, the prevalence of A/untyped (40%) increased considerably in 2020. In quail, however, the prevalence of A/H9 (56%) significantly increased in 2020. The mixed-effect logistic regression model showed that the vendors having waterfowl (AOR: 2.13; 95% CI: 1.04–4.33), purchasing birds from the wholesale market (AOR: 2.96; 95% CI: 1.48–5.92) instead of farms, mixing sick birds with the healthy ones (AOR: 1.60; 95% CI: 1.04–2.45) and mingling unsold birds with new birds (AOR: 3.07; 95% CI: 2.01–4.70) were significantly more likely to be positive for AIV compared with vendors that did not have these characteristics. We also found that the odds of AIV were more than twice as high in quail (AOR: 2.57; 95% CI: 1.61–4.11) as in pigeons. Furthermore, the likelihood of AIV detection was 4.19 times higher in sick and dead birds (95% CI: 2.38–7.35) than in healthy birds. Our study revealed that proper hygienic practices at the vendors in LBM are not maintained. We recommend improving biosecurity practices at the vendor level in LBM to limit the risk of AIV infection in pigeons and quail in Bangladesh.

## Introduction

The avian influenza virus (AIV) is a severe threat to Bangladesh's poultry sector, with substantial consequences for the economy and public health. It has also been identified in wild birds in Bangladesh, mainly crows (1, 2). Since the initial outbreak in poultry in 2007, H5N1 has posed public health and economic danger in Bangladesh. The virus has become enzootic in poultry, with 585 outbreaks documented in 54 of the 64 districts, making the country one with the most significant outbreaks globally (3, 4). On the other hand, since 2006, the H9N2 subtype has been the most common, followed by the highly pathogenic avian influenza (HPAI) H5N1 subtype, which has been detected from domestic land-based poultry in Bangladesh (5–8). H5N1 and H9N2 AIV have become endemic in poultry and have been observed sporadically infecting humans (9). Eight cases of H5N1 with one mortality and three cases of H9N2 have also been reported in Bangladesh (4, 9, 10). There is also a significant cause for concern regarding the co-infection of HPAI H5N1 and LPAI viruses, particularly H9N2.

In Bangladesh couple of steps have been taken to control the AIV, including the development of the national avian influenza and human pandemic influenza preparedness and response plan 2006–2008, which facilitate a co-coordinated and effective national response in the event of an incursion of HPAI/H5N1 in domestic poultry, and to minimize the risk of human pandemic influenza (HPI) (9). Isolation of HPAIV-infected flocks has relatively lower mortality and stamping out when mortality is higher (9). Official reporting to the Department of Livestock Services (DLS) is practiced in the latter case. Vaccination of poultry reduces the shedding of viruses, thereby decreasing the amount of viruses in the environment and at the poultry-human interface (11, 12). Multiple sporadic and discontinued AIV surveillance. Adopting essential biosecurity measures at the farm level and nationwide public awareness-raising efforts.

Most of the studies on AIV in Bangladesh have focused on chicken and ducks, but pigeons and quail are now being farmed in Bangladesh and are in danger of contracting the virus. The poultry industry is the most efficient and cost-effective source of animal protein, but rising future demand prohibits it from addressing the supply-demand gap for animal protein (10). Along with Contemporary broiler and layer, customers continually seek other safe meat options such as pigeons and quail (13). As a result, pigeons and quail raising has emerged to meet public demand and become economically prosperous.

Pigeons and quail farming is now a thriving industry in Bangladesh. Domestic pigeons have been raised for meat in Bangladesh for many years. According to a 2020 study, Bangladesh has a pigeon population of 10.8 million (14). As one of the most promising species for future income-earning options for many people, pigeons rearing is being investigated further to reduce Bangladesh's unemployment rate. Pigeons reproduce prolifically, and squab meat is in high demand in the market because of its delicacy and flavor (10).

On the other hand, quails are now utilized to produce commercial eggs and meat (13). They are the most suitable and effective birds for economic and nutritional purposes because they achieve sexual maturity quickly, have a shorter incubation time, and produce up to four generations yearly (15). Rearing quails can also boost nutritional value because quail meat has more protein than chicken (16). Quail farming began in Bangladesh in 1990, but it is now a thriving industry due to its economic importance as a commercially farmable species that produces excellent meats with delicate flavors (17). In recent years, native chickens and ducks, domestic pigeons, and quails have emerged as small-scale commercial ventures and demonstrated viability as a source of revenue for rural farmers under conventional management structures. The supply of pigeons and quail has expanded in the LBMs of Bangladesh due to the significant and rising demand for pigeons and quail meats and eggs. They are also kept as pet birds. Many vendors nowadays keep pigeons and quails in their stores; however, these vendors often lack the knowledge of biosecurity protocols necessary to maintain a healthy population of pigeons and quails, which puts the birds at risk of contracting AIV.

Pigeons and quails sold at live bird markets in countries such as Egypt, China, Indonesia, and Vietnam have previously been infected with the AIVs (18–20). Pigeons and quails in Bangladeshi LBMs have also been infected with the AIV virus (21). We should take appropriate measures to control the infection of the virus in these birds in the LBMs. However, to the best of our knowledge, there have not been many comprehensive studies that have explicitly targeted the circulation of AIV in pigeons and quails in the LBMs of Bangladesh, nor has there been an investigation into the risk factors that lead to the infection. As a result, we conducted this study to determine the prevalence of AIV and its subtypes in pigeons and quails in the LBMs in Bangladesh and determine the factors associated with AIV infection.

## Materials and methods

### Study sites, period, and design

**Figure 1:**
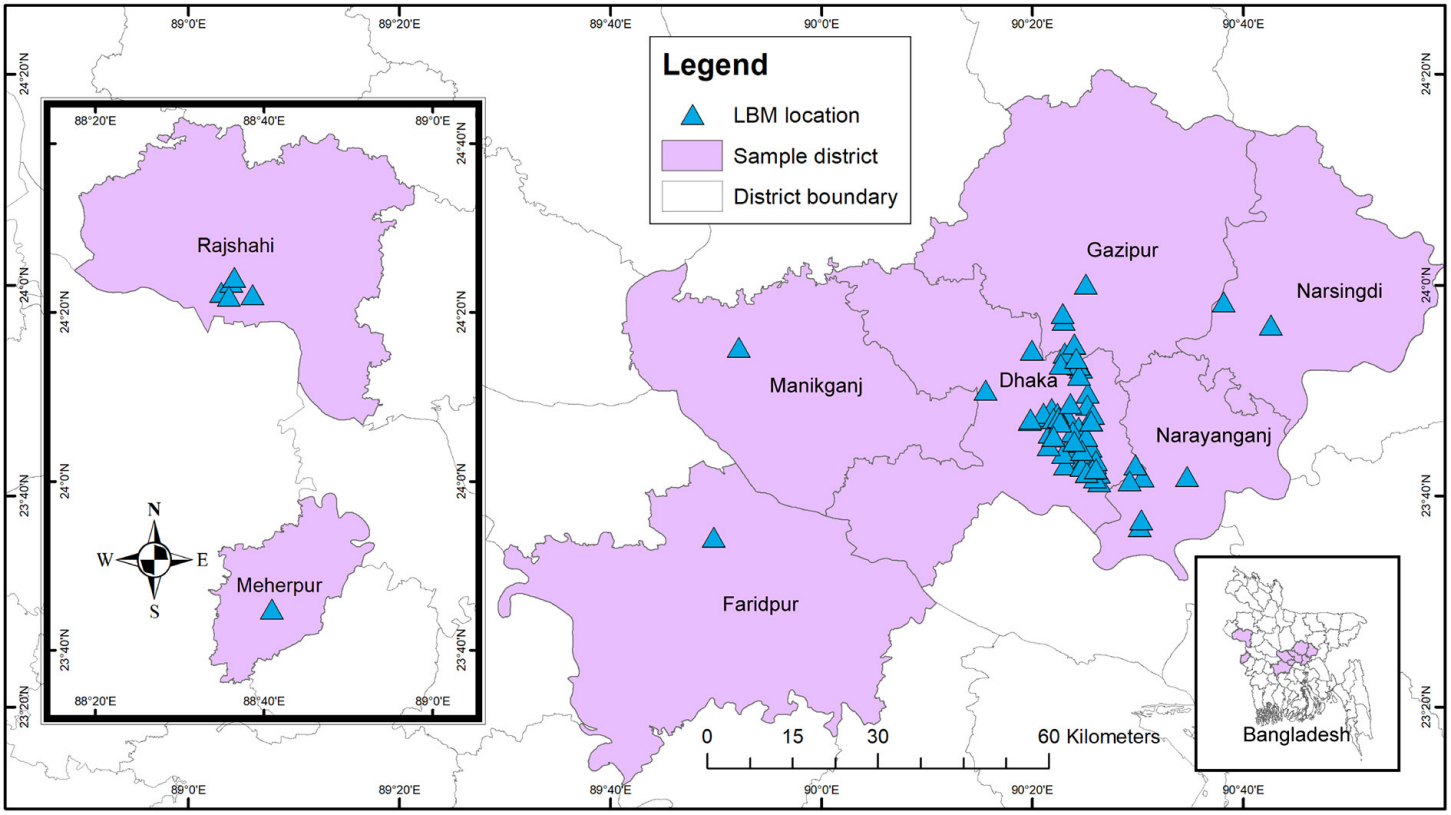
Sampling locations of LBMs in selective districts in Bangladesh.

We conducted a cross-sectional study on pigeons and quail in LBMs and the bio-security practices maintained in the vendor shop level and LBMs in 8 districts (Figure 1) from February 2017 to September 2021. We purposively selected the study areas based on the poultry and LBM density.

### Ethical approval

The study protocol was approved by the Animal Experimentation Ethics Committee of [protocol: CVASU/Dir (R&E) AEEC/2015/751] and Ethics Committee (EC) (Protocol: CVASU/Dir (R&E) EC/2015/1011) of the Chattogram Veterinary and Animal Sciences University.

### Biological samples and data collection

We collected pooled cloacal and oropharyngeal swab samples from each bird using two sterile cotton swabs and placed them in a 1.8 ml sterile cryovial containing 1 ml of viral transport media (VTM) sample as previously described (22). However, we are aware that some pet birds are very costly, and the owner is reluctant to provide cloacal or oropharyngeal swabs. In that case, we collected swabs from freshly laid feces. The number of collected samples from pigeons and quail during each year and season from LBMs in our studied locations are presented in Supplementary Figure S1.

Immediately after collection, samples were stored in liquid nitrogen in the field and at −80°C in the lab until laboratory testing.

We collected the necessary information for individual vendors, bird health conditions, and selling practices related to hygiene and sanitation using a structured multiple-choice questionnaire.

### Lab testing

We tested the swab samples to detect the viral M gene for the presence of AIV. The MagMAXTM-96 AI/ND Viral RNA Isolation Kit (Applied BiosystemsTM, San Francisco, CA) extracted RNA from collected samples in a KingFisherTM Flex 96-well robot (Thermo ScientificTM, Waltham, MA) according to the manufacturer's instructions. The samples were first tested for the presence of the M gene using real-time reverse transcription-polymerase chain reaction (rRT-PCR) with reference primers and probes, followed by the procedure as reported by Spackman (23). The H5, H9, and H7 sub-typing of M gene-positive samples were then determined utilizing primers and probes in an rRT-PCR test (24), followed by Spackman and Suarez (25). An example was considered positive if the cycle threshold value was <40 (26). Among M gene-positive samples, those negative for H5, H9, and H7 were considered Influenza A HA/untyped.

### Statistical analysis

We summarized the characteristics of biosecurity practices by using descriptive analyses. We then estimated the prevalence of influenza A viruses in different species and seasons, along with 95% CIs and visualized them using graphical analysis. We used a time plot of the prevalence of AIV subtypes to show the temporal trend of AIV subtypes. We then performed Pearson's chi-square test (27) to find the bio-security practices significantly associated with AIV. Factors associated with AIV with *p* < 0.05 in univariate analysis were selected for multivariable analyses. We then calculated Cramer's V to identify the relationship between the predictor variables. We then used a mixed-effect logistic regression model (28), accounting for clustering by district and live bird market, to estimate adjusted odds ratios. We calculated model χ^2^ to measure model fitness for the mixed-effect logistic regression model by Wald's test. We performed all statistical analyses using Stata version 16 software (StataCorp LLC, https://www.stata.com) and RStudio version 4.1.2 (29). We used “lme4” and “tidyverse” packages for the analysis in R software. For graphical presentation, GraphPad Prism (https://www.graphpad.com) and for mapping, ArcGIS (https://www.arcgis.com) were used. The shape file was collected from freely available DIVA-GIS (https://www.diva-gis.org/gdata).

## Results

### Assessment of the hygienic status of vendor level and live bird market level

The hygienic and sanitation status of the vendor and LBM are presented in Table 1. Sixty-four percent of the LBM had closing days, and more than eighty-six percent of the LBMs had more than one bird species (Chicken, duck, pigeon, quail). The presence of wild birds (93.29%) and waterfowl (59.27%) was noticed in most LBM and vendor stalls, respectively. On the other hand, pigeons were found traded at more vendor stalls than quails (38.34%). Over eighty percent of the vendors buy birds from the wholesale market and get them through inter-district trading. Most (62.94%) of the vendors do not separate the sick birds from the healthy birds, but they (72.36%) do not usually mix the unsold birds with the new birds. When it comes to the disposal of offal and dead birds, most vendors (68.89%) throw them away.

**Table 1:** Proportion and 95% CI of biosecurity and hygienic status of pigeons and quail vendor stall level and live bird markets level.

**Factor**	**Frequency (%)**	**95% CI**
**Presence of wild birds**		
No	42 (6.71)	4.99–8.96
Yes	584 (93.29)	91.04–95.01
**Presence of waterfowl**		
No	255 (40.73)	36.94–44.64
Yes	371 (59.27)	55.36–63.06
**Type of species**		
Pigeon	386 (61.66)	57.78–65.40
Quail	240 (38.34)	34.60–42.22
**Source of birds of vendor**		
Wholesale market	506 (80.83)	77.55–83.73
Farms	120 (19.17)	16.27–22.45
**Where do you get your birds?**		
Inter-district	543 (86.74)	83.85–89.18
Local area	83 (13.26)	10.82–16.15
**Separate sick birds at the vendor**		
No	394 (62.94)	59.08–66.64
Yes	232 (37.06)	33.36–40.92
**Mix unsold birds with new birds in the same cage and sell**		
No	453 (72.36)	68.72–75.73
Yes	173 (27.64)	24.27–31.28
**Disposal of offal and dead birds**
Dustbin	114 (18.21)	15.37–21.44
Throw away (in bushes/ water bodies)	430 (68.69)	64.94–72.21
Both	82 (13.10)	10.67–15.98
**The bird died in the last seven days at the shop**		
No	542 (86.58)	83.67–89.04
Yes	84 (13.42)	10.96–16.33
**Selling no of species birds at LBM**		
More than one species	541 (86.42)	83.50–88.89
Single poultry species	85 (13.58)	11.11–16.50
**Type of business of vendor**		
Retail	174 (27.80)	24.42–31.44
Wholesale	3 (0.48)	0.15–1.48
Both	449 (71.73)	68.06–75.12
**Health conditions of birds**		
Healthy	554 (88.50)	85.75–90.78
Sick or dead	72 (11.50)	9.22–14.25

CI, confidence interval.

In contrast, only 13.42% of shops reported having dead birds within the past week. Retail and wholesale businesses made up the majority (71.73%) of the stalls. The preponderance of shops (88.50%) had healthy rather than sick birds.

### Prevalence of AIV and subtypes at the vendor level in pigeons and quail

**Figure 2:**
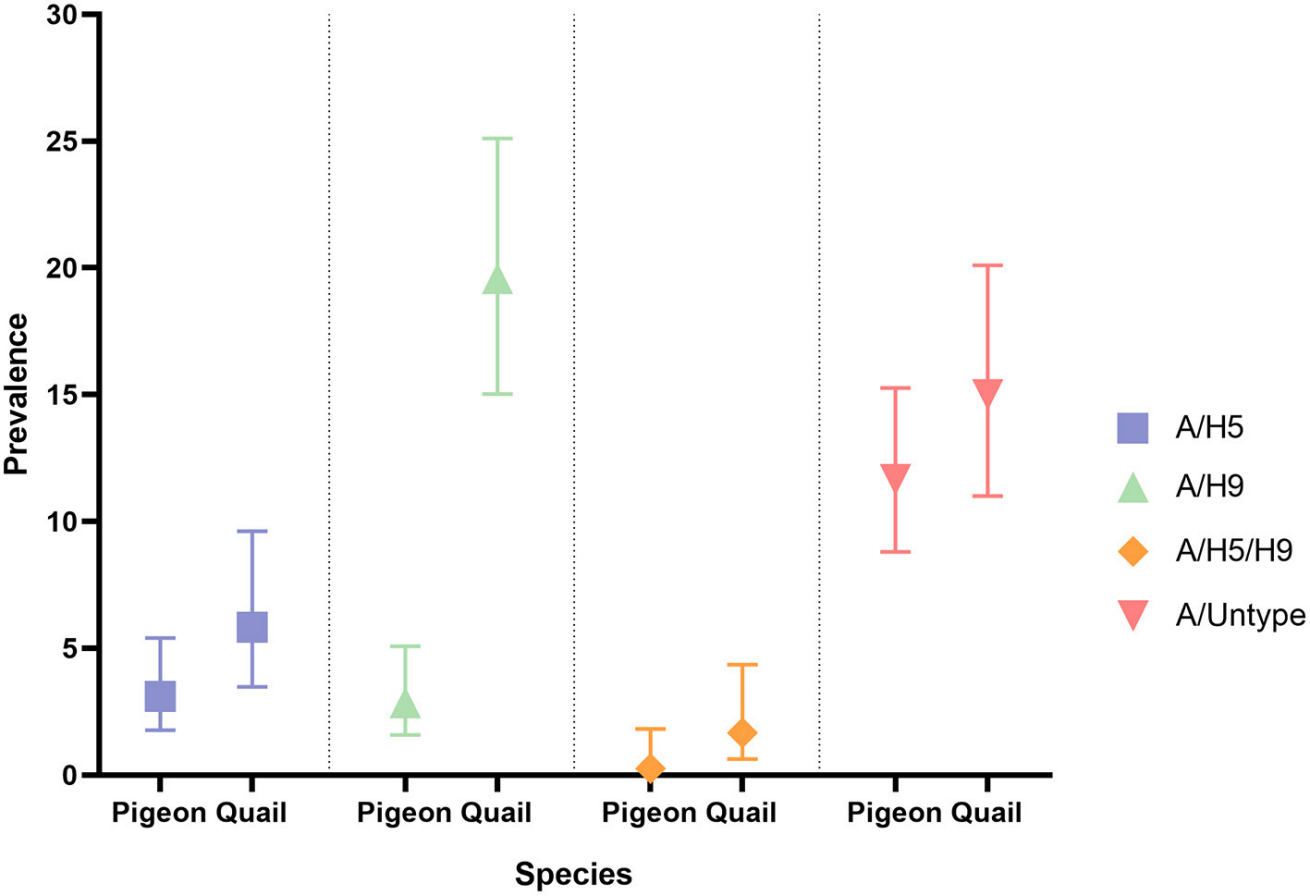
Prevalence and 95% CI of Influenza A subtypes in pigeons and quail.

Prevalence and 95% CI of Influenza A subtypes in pigeons and quail during 2017–2021 is presented in Figure 2.

The overall prevalence of AIV was 25.56%, A/H5 was 3.35%, and A/H9 was 8.47% in our sample. Co-circulation of H5 and H9 were also found in our sample (8%). We did not detect any H7 in our sample. Prevalence of AIV was higher in quail (38.75%) than in pigeons (17.36%). Similar results were also found for A/H5 (quail: 4.17% and pigeon: 2.85%), A/H9 (quail: 17.92% and pigeon: 2.59%) and A/H5/H9 (quail: 1.67% and pigeon: 0.26%).

### Seasonal pattern and temporal trend of prevalence of AIV and subtypes

**Figure 3:**
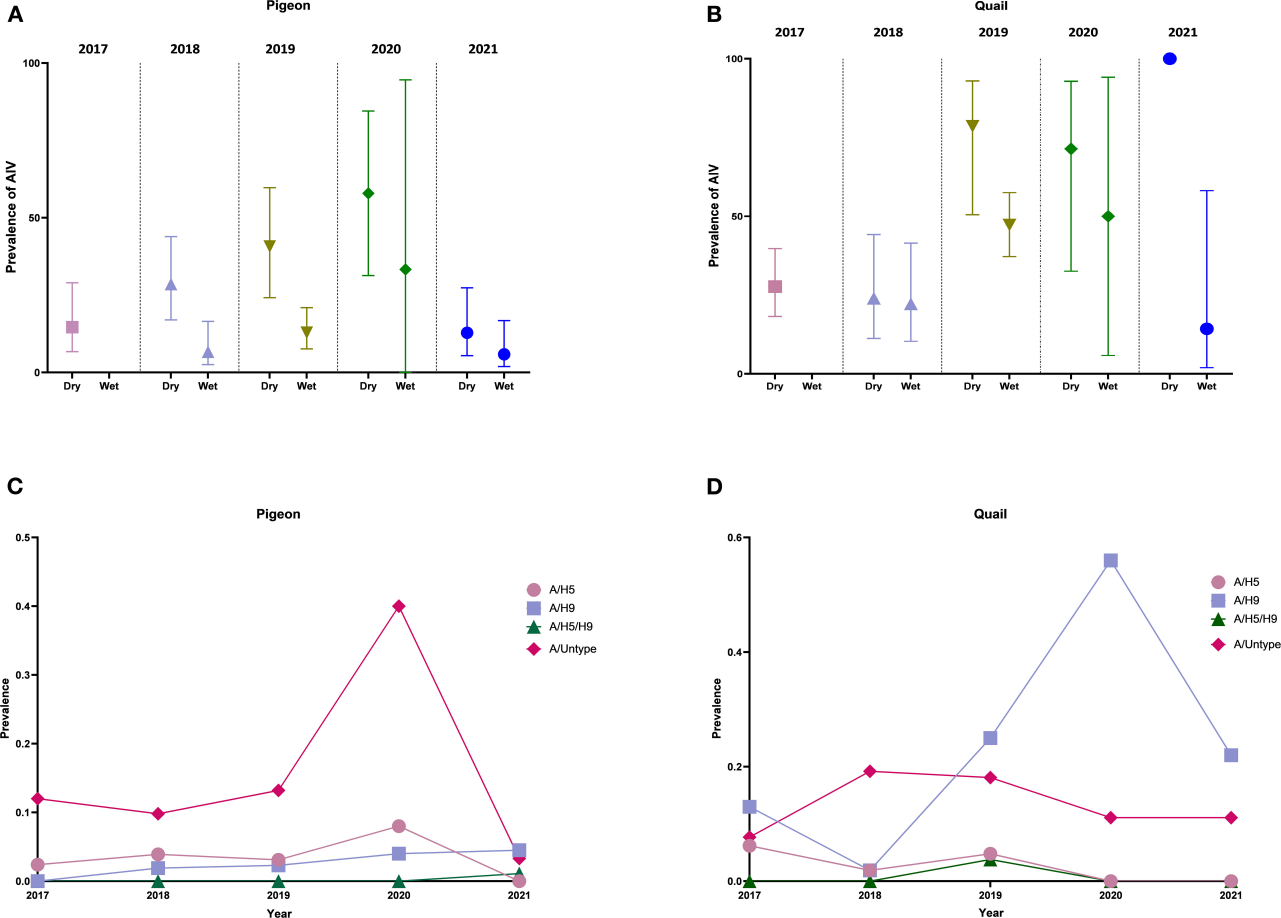
Seasonal pattern and temporal trend of prevalence of AIV and subtypes (2017–2021). **(A)** Prevalence and 95% Confidence Interval of AIV in pigeons in dry and wet seasons. **(B)** Prevalence and 95% Confidence Interval of AIV in quail in dry and wet seasons. **(C)** Temporal trend of AIV subtypes in pigeons from 2017 to 2021. **(D)** Temporal trend of AIV subtypes in quail from 2017 to 2021.

From Figures 3A,B, we can observe that for both pigeons and quail, there is a higher prevalence of AIV in the dry season every year than in the wet season. We also noticed the same seasonal pattern in the overall sample (Supplementary Figure S3). Figures 3C,D show the temporal trend of AIV subtypes in pigeons and quail from 2017 to 2021. In pigeons, we can see that, while the prevalence of A/H5, A/H9, and A/H5/H9 did not vary considerably between 2017 and 2021, the prevalence of A/untyped (40%) increased greatly in 2020. In quail, however, we detected no substantial shift in the prevalence of A/H5, A/untyped, and A/H5/H9 over the years, although the prevalence of A/H9 (56%) significantly increased in 2020. From Supplementary Figure S2, we can also see that in the dry season, the overall prevalence of A/H5 and A/H9 across all the years was higher than the overall prevalence of A/H5 and A/H9 in the wet season.

### Association between bio-security practices and AIV circulation in pigeons and quail

#### Univariable analysis to identify predictor variables using chi-square test

We used Pearson's chi-square test to determine the bio-security practices that influence the prevalence of AIV (Table 2). We only considered vendor-level bio-security practices for univariable analysis. In the univariate analysis, significant associations (*p* < 0.05) with AIV were found: the presence of waterfowl; type of species; source of birds of the vendor; source area of birds of the vendor; separate sick birds at the vendor; mixing unsold birds with new birds; history of any birds died in the last seven days at the shop; selling the number of species of birds; type of business of the vendor; and health conditions of birds.

**Table 2:** Factors associated with AIV circulation (results from Pearson's chi-square test ^*^p-value < 0.05, statistically significant).

	**Prevalence**	**95%**	***p*-**
	**(%)**	**confidence**	**value**
		**interval**	
**Presence of waterfowl**			
No	53 (20.78)	16.23–26.21	0.023*
Yes	107 (28.84)	24.45–33.67	
**Type of species**			
Pigeon	67 (17.36)	13.89–21.47	0.000*
Quail	93 (38.75)	32.78–45.08	
**Source of birds of vendor**			
Wholesale market	149 (29.45)	25.63–33.58	0.000*
Farms	11 (9.17)	5.14–15.81	
**Source area of birds of vendor**			
Inter district	150 (28.18)	24.54–32.12	0.000*
Local area	10 (8.43)	4.07–16.67	
**Separate sick birds at the vendor**			
No	112 (28.43)	24.18–33.09	0.032*
Yes	48 (20.69)	15.95–26.4	
**Mix unsold birds with new birds**			
No	91 (20.09)	16.64–24.04	0.000*
Yes	69 (39.88)	32.85–47.37	
**Disposal of offal and dead birds**			
Both dustbin and throw away	21 (25.61)	17.32–36.14	0.963
Dustbin	28 (24.56)	17.52–33.3	
Throw away (in bushes/water bodies)	111 (25.81)	21.89–30.17	
**The bird died in the last seven days at the shop**			
No	118 (21.77)	18.49–25.45	0.000*
Yes	42 (50)	39.45–60.55	
**Selling no. of species of birds**			
More than one species	126 (23.29)	19.91–27.05	0.001*
Single poultry species	34 (40)	30.15–50.73	
**Type of business of vendor**			
Mixed (retail and wholesale	134 (29.84)	25.78–34.25	0.000*
Retail	24 (13.79)	9.41–19.77	
Wholesale	2 (66.67)	15.29–95.68	
**Health conditions of birds**			
Healthy	121(21.84)	18.59–25.48	0.000*
Sick or dead	39(54.17)	42.62–65.28	

#### Exploring correlation between predictor variables to identify potential multicollinearity

**Figure 4:**
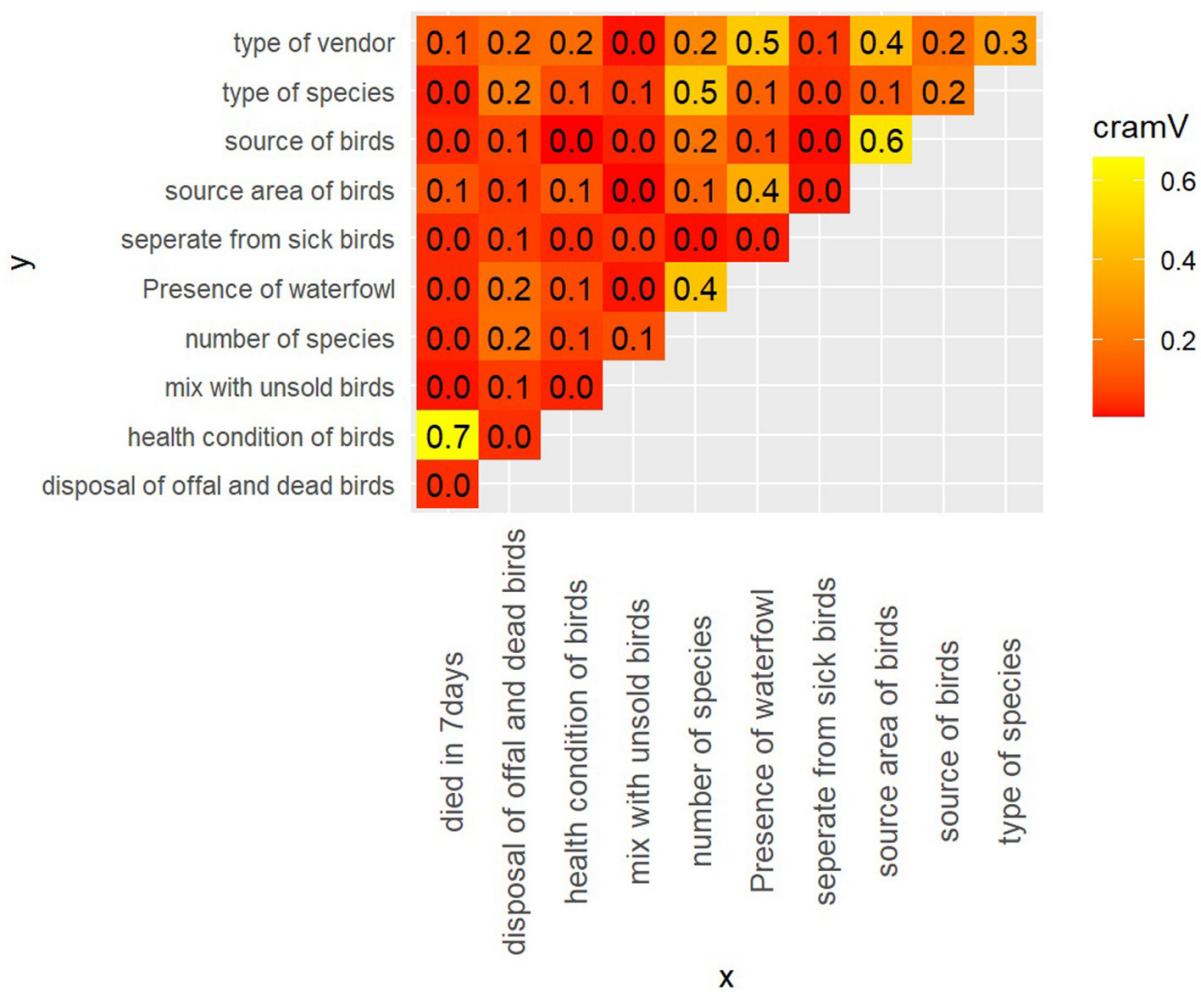
Value of Cramer's V between predictor variables.

The value of Cramer's *V* between the predictor variables is shown in Figure 4. Greater Cramer's *V* value suggests a stronger association. We chose 0.60 as the cutoff value (28). We can see a strong correlation between the health condition of the birds and the birds that died in the vendor shop in the last seven days (Cramer's *V* = 0.70). Similarly, the source of the birds of the vendors and the source area of the birds of the vendors were strongly correlated (Cramer's *V* = 0.60). To eliminate potential multi-collinearity, the source area of birds of vendors and birds that died in the last seven days at the shop will be omitted from the multivariable model.

#### Multivariable modeling using mixed effect logistic regression model

We used a mixed-effect logistic regression model, accounting for clustering by district and live birth market, to estimate the adjusted odds ratio (Table 3). In the multivariable analysis, we found that the presence of waterfowl, type of species (pigeons or quail), source of birds of the vendor, separate sick birds at the vendor, mixing of unsold birds with new birds, and health conditions of birds are the factors that have a significant association (*p* < 0.05) with AIV. Having waterfowl (AOR: 2.13; 95% CI: 1.04–4.33), Purchasing birds from the wholesale market (AOR: 2.96; 95% CI: 1.48–5.92) instead of farms, mixing sick birds with the healthy ones (AOR: 1.60; 95% CI: 1.04–2.45) and mixing unsold birds with new birds (AOR: 3.07; 95% CI: 2.01–4.70) were significantly more likely to be positive for influenza A viruses compared with vendors that did not have these characteristics. We also found that the odds of AIV were more than twice as high in quail (AOR: 2.57; 95% CI: 1.61–4.11) as in pigeons. Conversely, sick and dead birds had 4.19 times higher chance of AIV detection (AOR: 4.19; 95% CI: 2.38–7.35) compared to healthy birds. The mixed-effect logistic model fit our data well, with a chi-square value: of 87.68 and a *p*-value < 0.001.

**Table 3:** Bio-security practices associated with AIV circulation (results from mixed-effect logistic regression model)^T^, ^†^.

	**Odds ratio (95% CI)**	***p*-value**
**Presence of waterfowl**		
No	Reference	
Yes	2.13 (1.04–4.33)	0.04^*^
**Type of species**		
Pigeon	Reference	
Quail	2.49 (1.56–3.96)	<0.01^*^
**Source of birds of vendor**		
Farms	Reference	
Wholesale market	2.96 (1.48–5.92)	<0.01^*^
**Separate sick birds at the vendor**		
Yes	Reference	
No	1.60 (1.04–2.45)	0.03^*^
**Mix unsold birds with new birds in the same cage and sell**		
No	Reference	
Yes	3.07 (2.01–4.70)	<0.01^*^
**Selling no. of species birds**		
More than one species	Reference	
Single poultry species	1.51 (0.65–3.51)	0.34
**Type of business of vendor**		
Retail	Reference	
Mixed (retail and wholesale)	1.00 (0.49–2.04)	0.99
Wholesale	2.103 (0.15–21.88)	0.58
**Health conditions of birds**		
Healthy	Reference	
Sick or dead	4.19 (2.38–7.35)	<0.01^*^

T Variables for district and live bird markets were adjusted to account for clustering effects in multivariable analysis.

† Fitness of the mixed effect logistic regression model: chi-square value 87.68, p-value < 0.001.

* p-value < 0.05, significant variable.

## Discussion

Avian influenza viruses have caused devastating epidemics in domestic poultry and human infections in Bangladesh. AIV has also been detected in pet birds such as pigeons and quail in Bangladesh (19), but there have not been many studies identifying the variables that increase the risk of AIV in pigeons and quail. Our analysis depicts existing biosecurity practices in Bangladesh's vendor shops in selected LBMs. We studied the trends in AIV infections in pigeons and quail from 2017 to 2021. We identified certain biosecurity practices associated with the circulation of AIVs in pigeons and quail.

### Prevalence of AIV and subtypes in pigeon and quail

In the present study, the prevalence of AIV RNA in pigeons was 17.36%, higher than the prevalence previously observed in Bangladesh (21, 30). It may be because we collected extensive data from pigeons through intensive sampling, where they collected data from other birds along with pigeons and quail. In pigeons, we detected A/H5, A/H9, and co-circulation of A/H5/H9; similarly, prior investigations detected A/H5 and A/H9 (21). The prevalence of A/untyped was higher than A/H5, A/H9, and A/H5/H9 in pigeons suggesting other LPAIV subtypes are circulating among pigeons in the LBMs of Bangladesh. There is evidence that pigeons could be infected by LPAIV subtypes like H3N6 (31).

We detected that the prevalence of AIV in quails is 38.75%, which is comparable to the prevalence of AIV in the LBM (32) but higher than the prevalence identified in the Pet bird market (PBM) (21) in earlier studies on quails in Bangladesh. LBMs are considered hotspots for the occurrence and contamination of AIV (30). Therefore, our study's prevalence of quail was higher as we obtained data from LBMs. Similar to our research, AIV subtype H5, H9, and co-circulation of H5 and H9 were also detected in quail in the previous studies (4, 6). We found that the prevalence of A/H9 was higher in quail than in other subtypes. It has been found that quails are highly susceptible to H9N2 viruses, with the HA gene requiring few modifications for efficient replication and transmission in the quail (33). Unless other pathogens complicate the infection, birds infected with H9N2 AIV often exhibit no clinical symptoms or minor respiratory symptoms and a decline in egg production; hence, the virus remains unreported and spreads more rapidly than other subtypes (34).

We found that the prevalence of AIV in quail was more significant than in pigeons. AIVs do not replicate well in pigeons, which only shed a trace amount of the virus (33) with little or no clinical symptoms (33, 35). On the other hand, gallinaceous poultry, such as quail, is thought to be highly susceptible to AIV infection, resulting in significant morbidity, mortality, and gross and histological lesions (35). Also, quails are smaller in size, allowing more birds to be caged together; they are more vulnerable to influenza infection and have been linked to the land-based transmission and adaptation of H5 and H9 viruses in other hosts (33).

### Seasonal pattern and temporal trend of prevalence of AIV subtypes in pigeons and quail

According to the findings of our study, the incidence of AIV in both pigeons and quails is higher during the dry season. We observed this pattern to be consistent every year from 2017 to 2021. It is possible because of the dry season's low temperatures and low humidity (36). During the dry season, the average temperature in Bangladesh stays between 18 and 22°C, and during the wet season, the average temperature stays between 23 and 30°C (36). Hassan, Hoque (30) showed that the prevalence of AIV increased in colder winter months. A study examined how long the Indian H5N1 HPAI virus could survive in dry and wet poultry feces at 42, 37, 24, and 4 °C. They discovered that the virus could survive for long periods in the feces at low temperatures and could potentially act as a long-term source of influenza virus in the environment (37).

We then showed that the circulation of A/untyped increased in pigeons in 2020, followed by a reduction in 2021, and the circulation of A/H9 in quail increased during 2019–2020, followed by a decrease in 2021. The increase in the prevalence of A/untyped and A/H9 during 2019 and 2020 in pigeons and quail, respectively, can be due to the unexpected emergence of the COVID-19 pandemic; LBMs in several cities throughout the country were forced to shut immediately; highways were also blocked, logistics were hindered; socially concentrated activities were canceled; consumer demand decreased, and poultries in LBM specifically birds like pigeons and quail could not be sold. Because of the large number of live birds kept together, the virus spread rapidly among the birds. Similar results were also found by Guo, Song (38) in LBMs in China. On the other hand, in 2020, the Government of Bangladesh approved an H9N2 vaccination (CEVAC NEW FLU H9K) (39). However, pet birds such as pigeons and quails are seldom vaccinated, and vaccination programs typically target layer and breeder chickens (40).

Nevertheless, pigeons and quail are kept close to chickens and ducks in LBMs. Consequently, the virus spread from poultry to pigeons and quails might have decreased. Vaccination of poultry may thus have a beneficial impact on the spread of AIV in pigeons and quails. Therefore, the decline in the prevalence of A/H9 in 2021 in quails may be attributable to the vaccination campaign.

### AIV virus and associated factors

Our study reveals a lack of hygienic and biosecurity practices in the majority of vendor shops and LBMs, such as the presence of wild birds and waterfowl, purchase of birds from wholesale markets rather than farms, acquisition of birds through inter-district trading, failure to separate sick birds from healthy birds and to throw away dead birds and offal instead of disposing of in the dustbin (Table 2). These practices have increased AIV in other countries (20, 41).

From multivariable analysis, we found that the presence of waterfowl in the vendor shop increases the risk of AIV in pigeons and quail. There is ample evidence that influenza viruses were spread from waterfowl to commercial poultry and pet birds (42). Ducks are regarded as the AIV virus's 'Trojan horse' (43–45). Infection in ducks may go unnoticed, even with HPAI, and it often manifests itself clinically only after the infection has spread or until quick and aggressive monitoring is performed (46). Furthermore, the LBM's vendors are congested and lack space (47). Different birds are kept together in a small space and mixed (47). If waterfowl, pigeons, and quail are sold in the same shop, the virus can easily spread from waterfowl to pigeons and quail.

We identified that purchasing pigeons and quail from a wholesale market raises the risk of AIV rather than buying from a farm. Wu, et al. (48) also showed that the transmission risk of AIVs gradually increases along the poultry supply chain from farms to wholesale markets in China. Most farms breed one or two species of birds, and different species of birds are rarely mingled when transported from the farm to the vendor shops because farms usually trade nearby (49). In wholesale marketplaces, on the other hand, different types of poultry are kept together. Furthermore, several species of birds are mingled together and maintained in crowded spaces while transported from wholesale markets to vendor shops, significantly increasing the danger of AIV infection (50).

Based on the findings of our study, we determined that mixing unsold birds with new birds considerably raises the probability of AIV infection. There is evidence that Poultry that has not been sold but is infected with the influenza virus could potentially infect new poultry. (41). Conversely, after overnight poultry keeping was declared illegal in China, the percentage of AIV virus isolates found in chickens dropped by 84% (51).

Our study revealed that vendors who do not separate sick from healthy birds have a higher risk of AIV. We also found that the chance of AIV detection is higher in sick or dead birds. Both the highly pathogenic avian influenza H5N1 and the low-pathogenic avian influenza H9N2 have been linked to cases of illness in birds (52, 53). Furthermore, it is possible that the HPAI AIV was the cause of the birds' deaths (54). Therefore, the presence of sick and dead birds may indicate that the birds are infected with AIV, and not separating them from healthy birds causes the virus to disseminate to healthy birds, thereby increasing the risk of infection with AIV.

## Conclusion and recommendation

We found that circulation of AIV in pigeons and quail in LBM of Bangladesh was positively associated with the presence of waterfowl, purchasing a bird from the wholesale market, mixing sick birds with healthy ones, mixing unsold with new birds, keeping the sick or dead bird, having quails in the shop. It is possible that improving these biosecurity practices could prevent the spread of AIV. Our study revealed that purchasing pigeons and quail directly from farms rather than wholesale markets was associated with a lower risk of AIV. Therefore, to understand the stages of the AIV subtypes infection in pigeons and quails, we should conduct a comprehensive investigation to determine what stage of the birds' distribution chain becomes infected and what factors contribute to this concern. Our research findings showed that proper biosecurity practices and hygiene standards are not maintained in the LBM in Bangladesh. Training for poultry workers on effective biosecurity practices to reduce the risk of AIV contamination should be implemented. Suitable vaccination programs targeting pigeons and quails must be carried out precisely to lessen the likelihood of AIV.

## Data availability statement

The raw data supporting the conclusions of this article will be made available by the authors, without undue reservation.

## Ethics statement

We performed all procedures in studies in accordance with the Ethical Standards of the Institutional and National Research Committee and with the 1964 Helsinki Declaration and its later amendments or comparable ethical standards. As part of standard Ethics approval procedures, participants were informed about the purpose, methods, risks, and benefits of participating in the study and were allowed to ask questions before providing voluntary consent. We obtained informed consent from all study participants before conducting interviews. The study protocol was approved by the Ethics Committee (EC) (Protocol: CVASU/Dir(R&E) EC/2015/1011) of the Chattogram Veterinary and Animal Sciences University. The animal study was reviewed and approved by the Chattogram Veterinary and Animal Sciences University-Animal Experimentation Ethics Committee (Protocol: CVASU/Dir (R&E) AEEC/2015/751).

## Author contributions

AI: conceptualization. AI and SI: field investigation. AI, MEH, and SI: data curation. RH, MS, MEH, and MR: laboratory analysis. AI and EA: formal analysis and wrote initial draft. AI and MR: funding. MMH and MR: supervision. SI and MMH: review and edited manuscript. All authors have read and approved the final version of the manuscript.

## Conflict of interest

AI was employed by EcoHealth Alliance. The remaining authors declare that the research was conducted in the absence of any commercial or financial relationships that could be construed as a potential conflict of interest.

## Publisher's note

All claims expressed in this article are solely those of the authors and do not necessarily represent those of their affiliated organizations, or those of the publisher, the editors and the reviewers. Any product that may be evaluated in this article, or claim that may be made by its manufacturer, is not guaranteed or endorsed by the publisher.
