# Supplementary figures and images for: Patterns and risk factors of avian influenza A(H5) and A(H9) virus infection in pigeons and quail at live bird markets in Bangladesh, 2017–2021
Source: Front Vet Sci. 2022 Oct 26;9:1016970. doi: 10.3389/fvets.2022.1016970 (PMC9645412; doi:10.3389/fvets.2022.1016970)

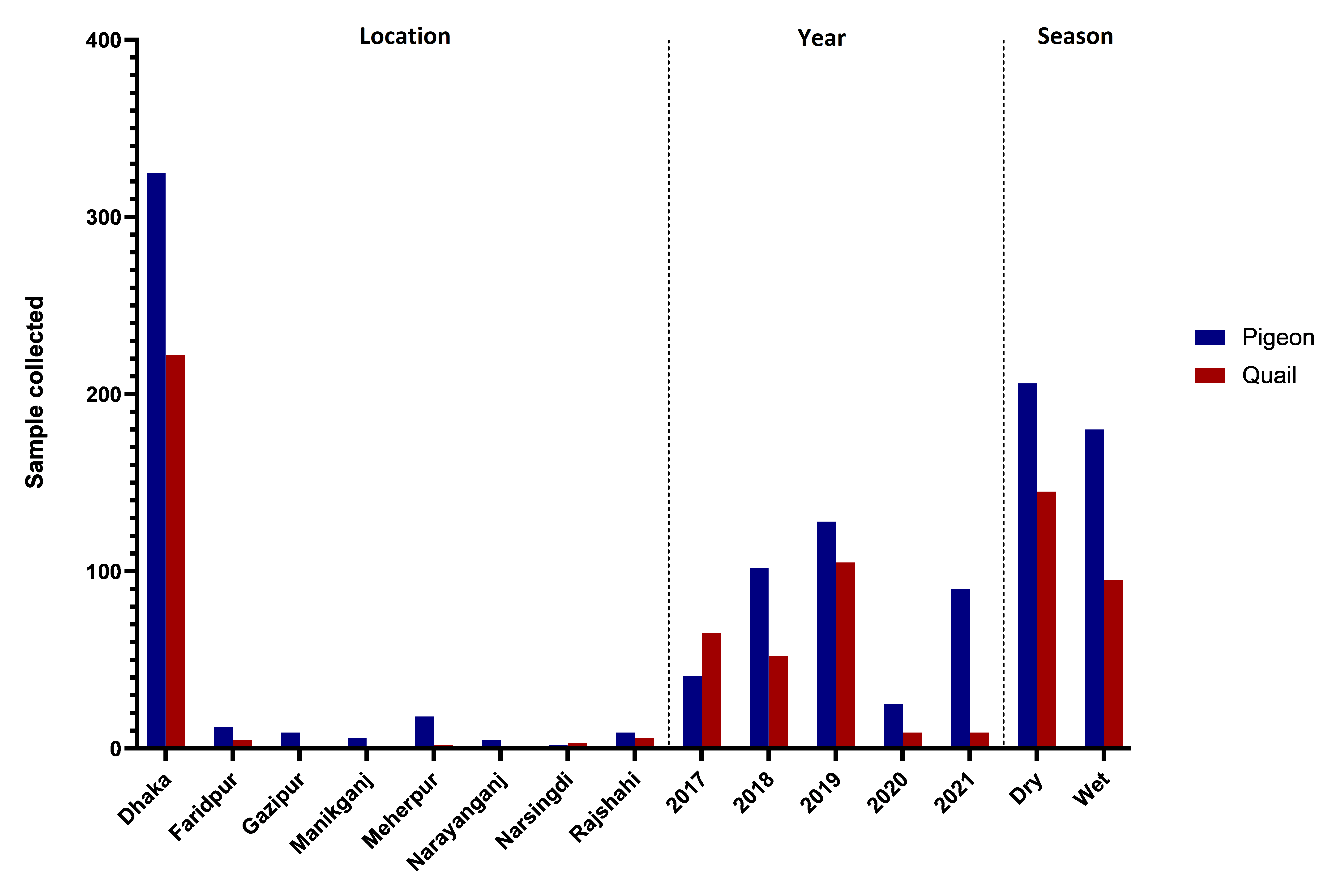

Supplement: Supplementary file 2 [file Image_1.TIF]

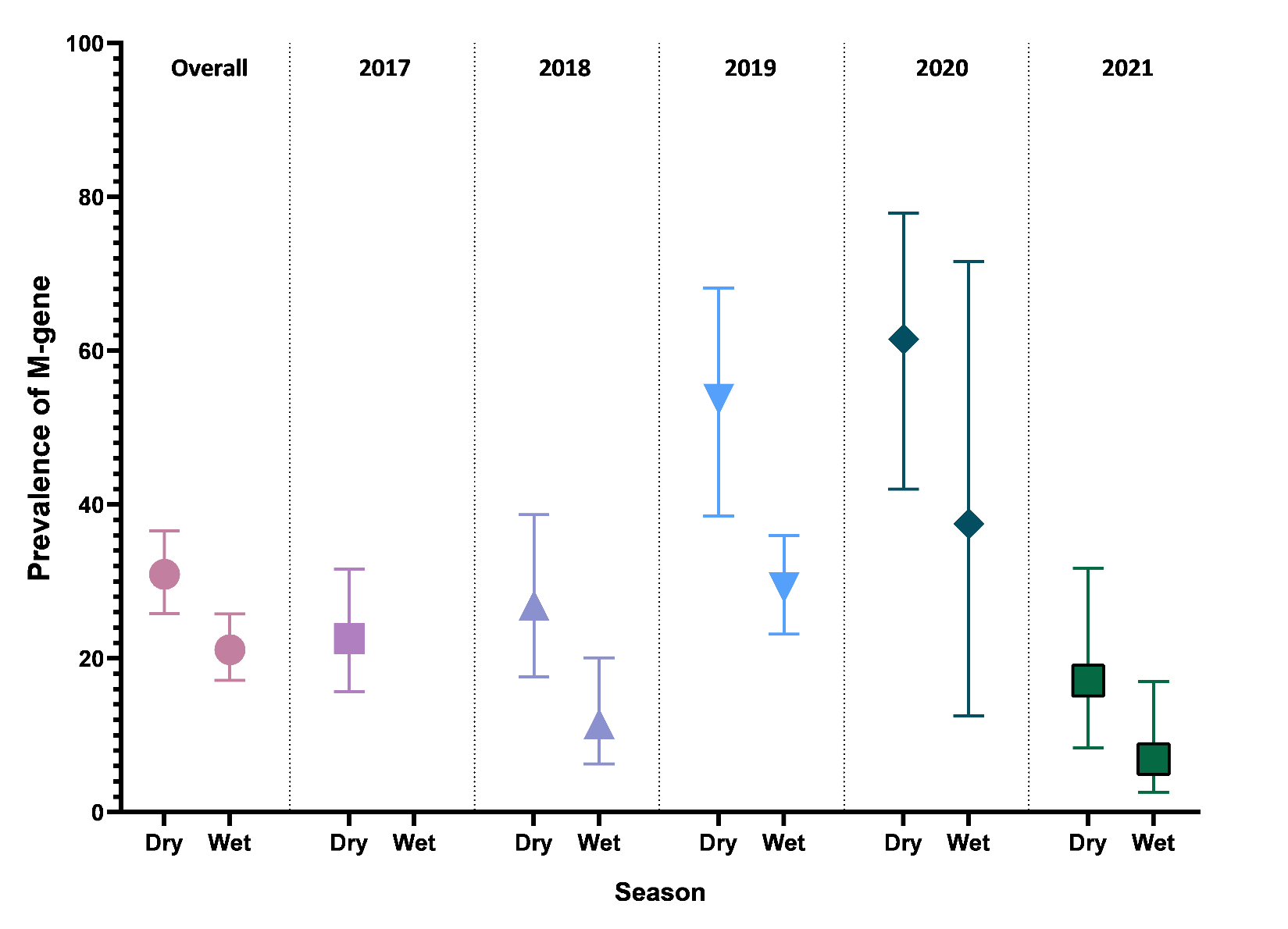

Supplement: Supplementary file 3 [file Image_2.TIF]

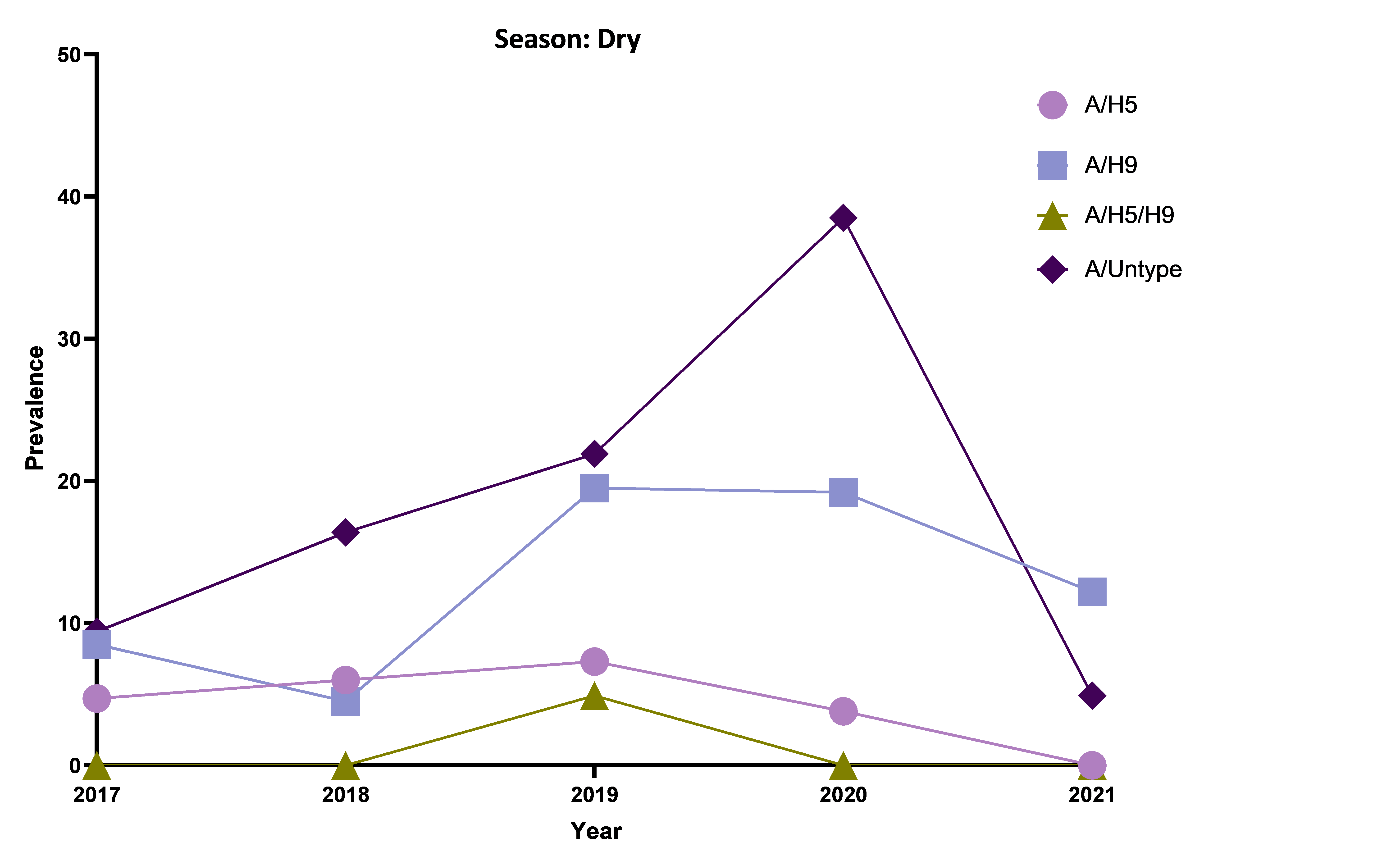

Supplement: Supplementary file 4 [file Image_3.TIF]

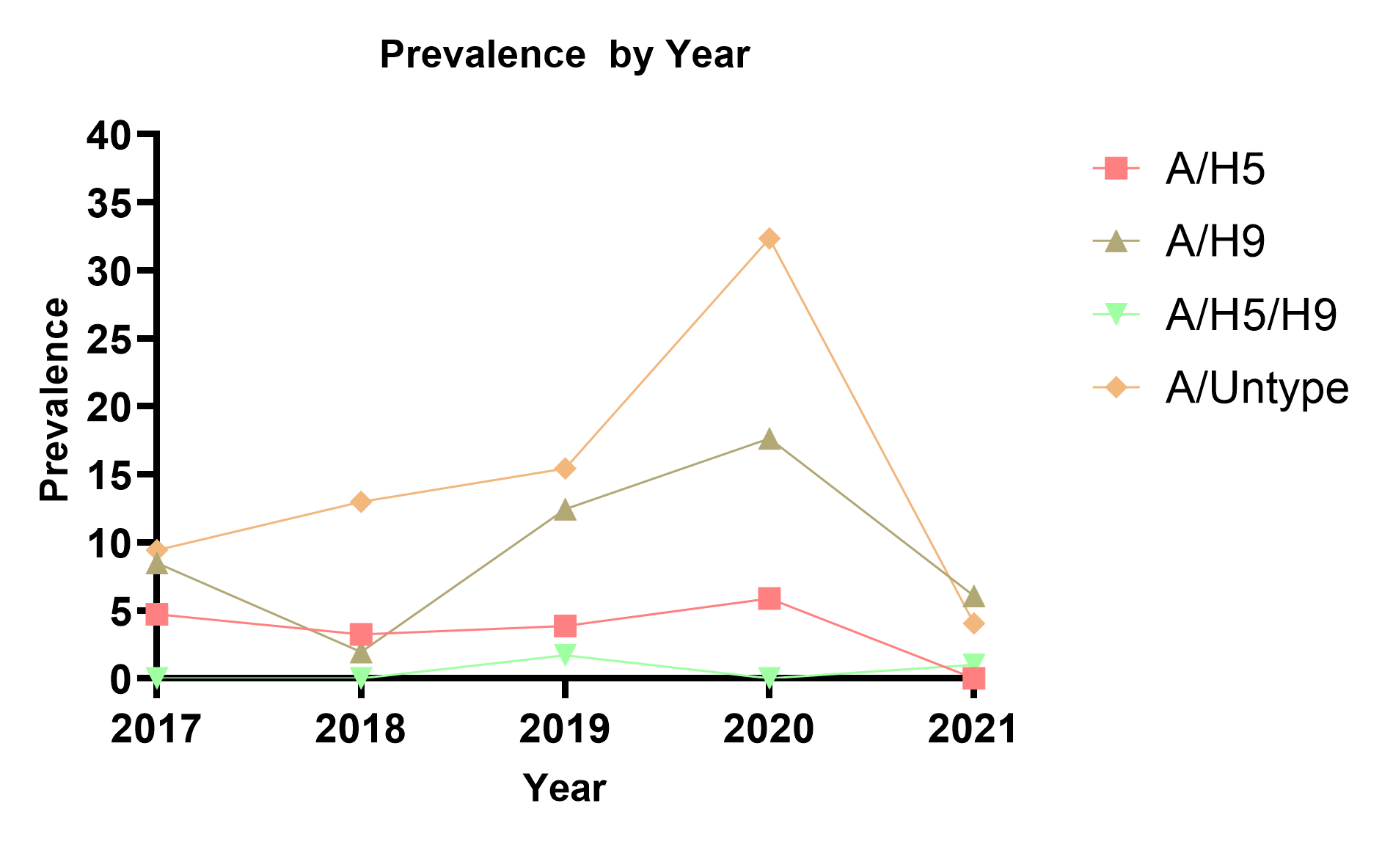

Supplement: Supplementary file 5 [file Image_4.TIF]

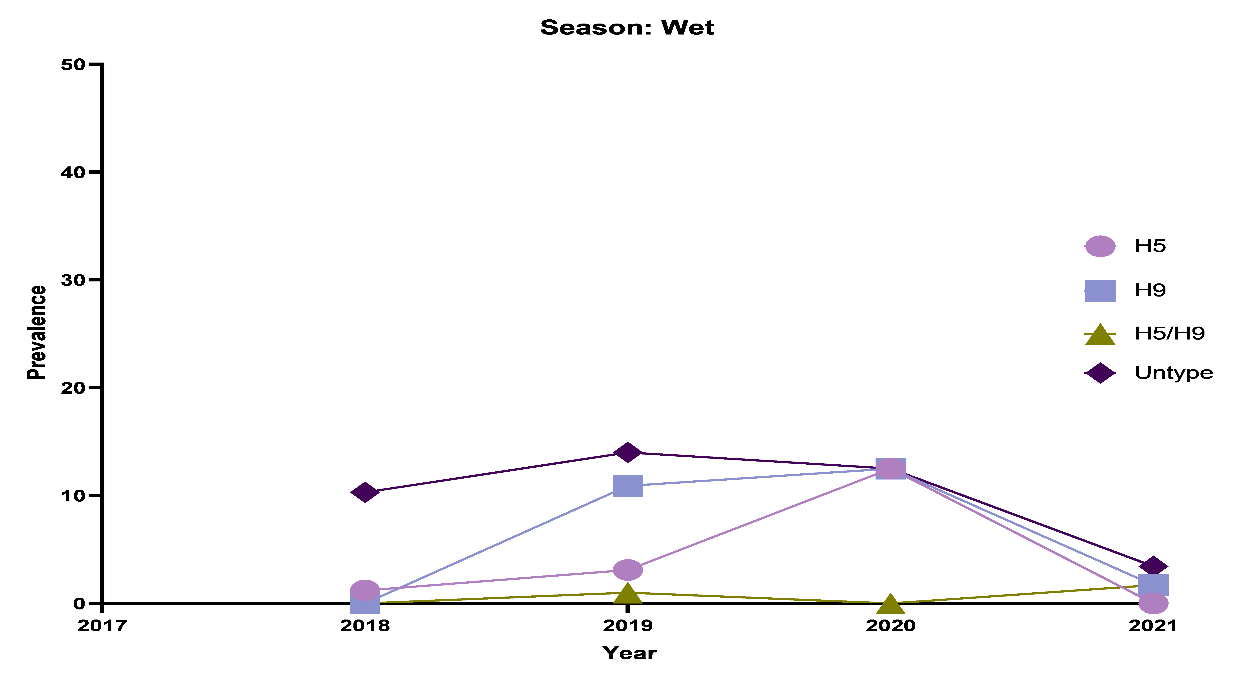

Supplement: Supplementary file 6 [file Image_5.TIF]
